# Indicators for Monitoring and Evaluation of Community-Based Injectable Contraception: Multisourced Process and New Global Guidance

**DOI:** 10.9745/GHSP-D-19-00133

**Published:** 2019-09-23

**Authors:** Jill M. Peterson, Kirsten Krueger, John Stanback

**Affiliations:** a FHI 360, Lilongwe, Malawi.; b FHI 360, Durham, NC, USA.

## Abstract

For many women, convenient access to family planning in their communities provides the impetus they need to start and maintain use of a family planning method. The benefits of family planning task shifting, which allows community health workers (CHWs) to provide methods such as oral contraceptive pills and injectables within communities, were publicly recognized by the World Health Organization (WHO) in 2009. WHO's 2012 global guidelines on task sharing recommended CHW provision of injectable contraception, accompanied by targeted monitoring and evaluation (M&E); however, the term “targeted” was not defined. To fill this gap, we undertook a literature review, technical consultation, and case studies in Malawi, Senegal, and Uganda to inform our development of a list of M&E indicators, supporting guidance, and job aids to help strengthen community-based access to injectable contraception programs and their ability to follow WHO recommendations. We identified 4 essential indicators: there are enough CHWs certified to provide injectables to meet project goals, CHWs are being appropriately supervised to ensure client safety, the stock of injectables is reliable and can meet project goals, and clients are receiving injections.

## Abstract (precis)

We based our guidance on a literature review, technical consultation, and case studies of 3 countries. We identified 4 essential indicators: enough community health workers (CHWs) certified to provide injectables to meet project goals, CHWs are appropriately supervised, stock of injectables is reliable, and clients are receiving injections.

## BACKGROUND

Increasing the use of family planning in developing countries is linked to reducing poverty, hunger, and maternal and childhood deaths. Consequently, it has been an important component of world development goals.^1^^–^^5^ For many women, convenient access to family planning in their communities provides the impetus they need to start and maintain use of a method. Numerous countries have thus implemented programs in which community health workers (CHWs), also known as lay health workers, counsel women in their communities on family planning and provide them with methods, allowing them to avoid the need to visit distant health facilities. In sub-Saharan Africa, this practice was initially most common for distribution of oral contraceptive pills and condoms, but in the mid-2000s, it began to include injectable contraception in several countries.^6^^,^^7^ Having recognized and promoted community-based access to injectable contraception (CBA2I) since 2009, the World Health Organization (WHO) included it in their written guidance in 2012.^8^^,^^9^ The document on optimizing health workers' roles focused on task sharing for maternal and newborn health and recommended that CHW provision of injectable contraception be accompanied by “targeted” monitoring and evaluation (M&E).^9^ However, the guidelines did not define what this particular type of M&E should entail. Today, more than a dozen countries have implemented CBA2I programs, including some of those with the poorest access to contraception, and approximately 10 others are exploring the possibility or have discussions or pilot programs underway.

To assist countries in following this WHO recommendation, we developed written guidance on M&E of CBA2I, including recommended M&E indicators. This article outlines the process we used to develop the guidance and indicators.

## METHODS

We developed the M&E guidance and indicators, using a systematic, 3-stage process that included a literature review, a technical consultation, and case studies.

We use a systematic, 3-stage process to develop the M&E guidance and indicators.

### Literature Review

Our literature review pertained to the status of CBA2I around the world, focusing on the M&E of CBA2I programs, including any currently used indicators. We searched the Global Health (produced by CABI), POPLINE, and PubMed databases for publications from the mid-1990s to April 2016, using search terms including “community + family planning,” “community + family planning + inject,” and “injectable contraception.” In addition to searching online databases, we included documents and tools from our own in-country CBA2I activities. We also asked colleagues from various partner organizations for relevant information. We compiled what we had learned by country and then did more intensive data gathering for the 13 countries for which we had relevant literature.

### Technical Consultation

We subsequently convened a group of 12 experts in the fields of family planning program implementation and M&E of family planning for a 2-day workshop. These technical experts were purposively selected to represent various organizations and regions of the world, as well as diverse country experiences in implementing CBA2I, in part based on findings from our literature review. The group included representation from government, international NGOs (INGOs), and WHO. At the workshop, we used a facilitated, consensus-building process to draft indicators.

The process began with participants sharing experiences with CBA2I and M&E of programs in the countries represented. Information gathered through the literature review was also shared. The group then discussed and documented how to reach key audiences, recognizing the importance not only of developing guidance and indicators, but also disseminating them.

Next, the group conceptualized a logic model for a hypothetical CBA2I program. The group agreed on typical CBA2I program outcomes and activities—key aspects of a logic model—which were then used to develop relevant indicators. The participants discussed indicators, created categories, and grouped indicators by category. They then divided into small groups and discussed indicators for the first category and later returned as a large group to revise the indicators until all suggestions and phrasing from the small groups were incorporated. This process was repeated for all indicator categories. The final result was 5 categories: training, supervision, readiness, service delivery, and data quality.

The discussion also included differentiating between “nice-to-have” indicators and indicators considered essential for a well-managed program. After the meeting, the authors led the effort to refine the indicators and invited the technical experts from the consultation to provide feedback on the revisions.

### Case Studies

To further explore the feasibility of the proposed indicators, we conducted case studies in Malawi, Senegal, and Uganda. We used data collected during the literature review to select these countries, which represented various regions of anglophone and francophone sub-Saharan Africa, a range of implementation models (national programs versus smaller-scale programs implemented by INGOs), varying education levels and training programs for the CHWs, and longstanding programs, as well as newer ones.

We developed standard interview questions, and in each country, we sought to interview the following:

- Parties responsible for administering the CBA2I program, including higher-level government officials in the family planning division
- District staff responsible for family planning
- Facility-based staff responsible for providing and/or overseeing family planning
- CHWs who were providing CBA2I
- Personnel at NGOs who played a role in establishing CBA2I projects and specifically the M&E of those projects

Through these interviews, we gained a better understanding of how CBA2I was managed, including the skills and training of CHWs and their other service responsibilities. We asked what indicators countries were currently using, and we consulted client registers and other tools and job aids as available. We probed to understand how the data contributed to decision making at a programmatic or national level. Lastly, we documented other data the countries wished they had to help them better manage and improve their programs.

To anticipate the utility and effectiveness of our indicators, we sought to understand how data were collected and compiled, which included asking ministry of health officials, M&E officers, and others with a role in analysis for estimates of the amount of time that typically elapsed between data collection and analysis. Lastly, we asked the interview respondents to identify best practices for M&E of CBA2I that they wanted to share with others who were initiating or expanding programs.

## RESULTS

By triangulating data from the literature review, technical consultation, and case studies, we produced an M&E guidelines document, an executive summary, and 3 case study summaries. The guidelines include a full list of 18 M&E indicators and indicator definitions. In addition, the guidelines propose programmatic recommendations and sample job aids to facilitate implementation of CBA2I. Programmatic recommendations include conducting regular supervision, providing training on data collection and use, ensuring timely and accurate reporting, analyzing data for use at multiple levels, conducting regular data quality assessments, and recognizing and supporting CHWs. The job aids provide sample forms for tracking service provision and commodities and are intended to be adopted by existing programs, as well as incorporated into new programs. Editable versions are available for customization by individual programs.

### Program Differences

The literature review provided useful information for understanding how CBA2I programs operate in various countries. For example, we learned about differences in the educational level and training requirements for the CHWs (some countries had literacy requirements for CHWs, others did not), restrictions on provision of injectables (some programs could only provide reinjections, not the initial dose), and implementation models (some were implemented nationwide by governments, others in programmatically supported districts). This background information helped us identify technical experts for the workshop and select countries for our case studies, and it allowed us to develop a deeper understanding of the current status of CBA2I in countries to ensure broad usability of the guidance.

### Essential Indicators

The guidelines contain the list of the 18 M&E indicators initially developed during the technical consultation process, 4 of which we have designated “essential.” Cognizant that collecting data on 18 indicators would be difficult for many programs, we agreed that by using the 4 essential indicators, CBA2I programs could get adequate feedback for good management. These indicators were deemed essential because they measure the process from beginning to end—whether there are enough CHWs certified to provide injectables to meet project goals, that CHWs are being appropriately supervised to ensure client safety, that the stock of injectables is reliable and can meet project goals, and that clients are receiving injections. The 4 essential indicators are as follows:

- **Number and percentage of CHWs certified to inject contraception:** Program managers should use this indicator to determine that the number of CHWs certified to inject contraception is sufficient to meet project goals. In addition, most of those trained should be officially certified within the program's regular certification time frame and process.
- **Number and percentage of CHWs certified during the previous reporting period who received at least 1 in-person supportive supervision visit for providing injectable contraception within [x] months after successful completion of practicum:** As we learned through our case studies, supervisory visits play an extremely important role in monitoring the safety and effectiveness of CBA2I programs. Although programs may vary in the intervals of supervisory visits, we recommend at least 1 visit per month in the first few months immediately following certification. After a CHW has proven to provide high-quality injectable services, the visits may be reduced to every quarter, which can be incorporated into existing processes.
- **Number and percentage of CHWs reporting a stock-out of injectables:** Just as programs need to ensure that enough CHWs are available, the workers must also have sufficient stocks of injectables to meet demand. Ideally, stock-outs should be rare or nonexistent, and any reports of regular stock-outs should be investigated immediately.
- **Number of injections provided by CHWs:** At a bare minimum, knowing the number of injections provided by CHWs can help program managers understand whether they have created enough demand for CBA2I services. Tracking the number of injections provided compared with program targets and past trends helps program managers identify concerns early.

By using the 4 essential indicators, CBA2I programs obtain adequate feedback of their process from beginning to end, enabling good management.

The 18 indicators (Table 1) also include measures of trainings held; providers demonstrating adequate skills during supervisory visits; geographic coverage; and whether data are timely, accurate, and reliable. In addition, because some countries have expressed concerns that this lay cadre of health workers lacks the skills to provide injections without harming clients, we included indicators covering problems of infection or accidental needle sticks. We termed these “reportable incidents,” recognizing that M&E should not be the first nor the only place these events should be reported. Rather, they should be reported immediately through clinical channels, specifically to a facility-based supervisor in most cases. After any immediate reports, M&E can be used to monitor the number and patterns of these incidents. For example, M&E would be effective in detecting if clusters of reportable incidents are occurring in certain geographic areas or if the number of incidents exceeds acceptable levels.

**TABLE 1.** Eighteen Recommended Monitoring and Evaluation Indicators for Community-based Access to Injectable Contraception Programs

Number	Indicator Definition	Numerator/Denominator
**Training**		
1.1	Number of CHWs trained in providing injectable contraception	NA
1.2	Number of CHWs who passed a post-training test on injectable contraception	NA
1.3 (n) 1.4 (%)	Number and percentage of CHWs certified to inject contraception^a^	Numerator: 1.3 Denominator: 1.1
1.5 (n) 1.6 (%)	Number and percentage of CHWs certified to provide injectable contraception who express confidence in their skills and abilities	Numerator: 1.5 Denominator: 1.3
1.7	Number of training courses held on community-based provision of injectable contraception	NA
**Supervision**		
2.1 (n) 2.2 (%)	Number and percentage of CHWs certified during the *previous* reporting period who received at least 1 in-person supportive supervision visit for providing injectable contraception within [x] months after successful completion of practicum^a^	Numerator: 2.1 Denominator: 1.3
2.3 (n) 2.4 (%)	Number and percentage of CHWs supervised in-person at least once within [x] months after successful completion of practicum *who demonstrated adequate skills at the time of first supervision*	Numerator: 2.3 Denominator: 2.1
**Readiness**		
3.1 (n) 3.2 (%)	Number and percentage of CHWs certified in providing injectable contraception who have given an injection in the last quarter	Numerator: 3.1 Denominator: 1.3
3.3 (n) 3.4 (%)	Number and percentage of villages/catchment areas with a CHW certified to provide injectable contraception	Numerator: 3.1 Denominator: Total number of villages/catchment areas
3.5	Number of households served per CHW	Numerator: Number of households in a catchment area in the reporting period Denominator: Total number of active CHWs in the catchment area in the reporting period
3.6 (n) 3.7 (%)	Number and percentage of CHWs reporting a stock-out of injectable^a^	Numerator: 3.6 Denominator: 3.1
**Service delivery**		
4.1	Number of CHW-led mobilization events	NA
4.2	Number of one-on-one family planning counseling sessions held by CHWs	NA
4.3	Number of injections provided^a^	NA
4.4	Number of reportable incidents including accidental needle sticks, or infections or abscesses at the site of the injection	NA
**Data quality**		
5.1 (n) 5.2 (%)	Number and percentage of CHWs submitting data reports on time	Numerator: 5.1 Denominator: 3.1
5.3 (n) 5.4 (%)	Number and percentage of CHWs submitting complete client data reports	Numerator: 5.3 Denominator: 3.1
5.5 (n) 5.6 (%)	Number and percentage of CHWs submitting reports with reasonable accurateness	Numerator: 5.5 Denominator: 3.1

Abbreviation: CHW, community health worker.

a Essential indicators.

In the indicators table, we have provided definitions and additional information, such as how calculations can be made, suggestions for disaggregation, and variation that can be expected by program or country. We do not propose data sources, because they will vary by country. Some countries have national programs and may be able to use data from the health management information system, but others may be subnational programs with data collected only by special forms at a programmatic level. Each country or program should establish the most accurate, reliable, and timely data source in its context.

### Variations in Program Implementation

We conducted 5 interviews in Malawi, 5 in Senegal, and 7 in Uganda. Through these interviews we noted variations in program implementation (Table 2). For example, Malawi's CBA2I program is implemented nationwide by a cadre of CHWs called health surveillance assistants who receive a regular salary. In Uganda, the program is implemented in certain INGO-supported districts—approximately one-third of all districts—and the CHWs are unpaid volunteers. In Senegal, the Department of Reproductive Health and Child Survival approved a policy to scale up CBA2I by trained traditional midwives, *matrones*, in 2014, but the supply of commodities has been inconsistent. The matrones are paid at the discretion of the local health sector management.

**TABLE 2.** Variations in Community-based Access to Injectable Contraception Programs, by Country

	Malawi	Senegal	Uganda
CHW cadre	Heath surveillance assistant	Agents de santé Communautaire and/*matrone*	Village heath teams
Education level	Must have a Malawi Certificate of Education, (equivalent of secondary school completion)	Must have minimum of a primary education	Must have completed at least 7 years of primary schooling
Training	10 weeks Injectables: 2 days theory; 3 days practicum	1-2 weeks Injectables: 3 days theory; 5 days practicum	1 week Injectables: 7-10 days
Payment	Approximately 100 USD/month	Unpaid	Unpaid
Program	Nationwide program, supported by Ministry of Health	Joint government/partner supported program	Partner-supported program

Abbreviation: CHW, community health worker.

Case-study countries were already collecting CBA2I-specific data, including CBA2I data disaggregated by method initiated, new or continuing users, and the number of commodities used. For example, the countries monitored CBA2I data on the number of new family planning users by method, the number of continuing family planning users by method, and the number of injections and oral contraceptive pills distributed during that reporting period. In Senegal and Uganda, the data are available by community-based provision, but in Malawi, injectables provided by CHWs are simply included in total tallies for the facility to which the CHW reports. As a result, analysis of injections given by CHWs, as opposed to facility-based cadres, is not possible in Malawi.

**Figure:**
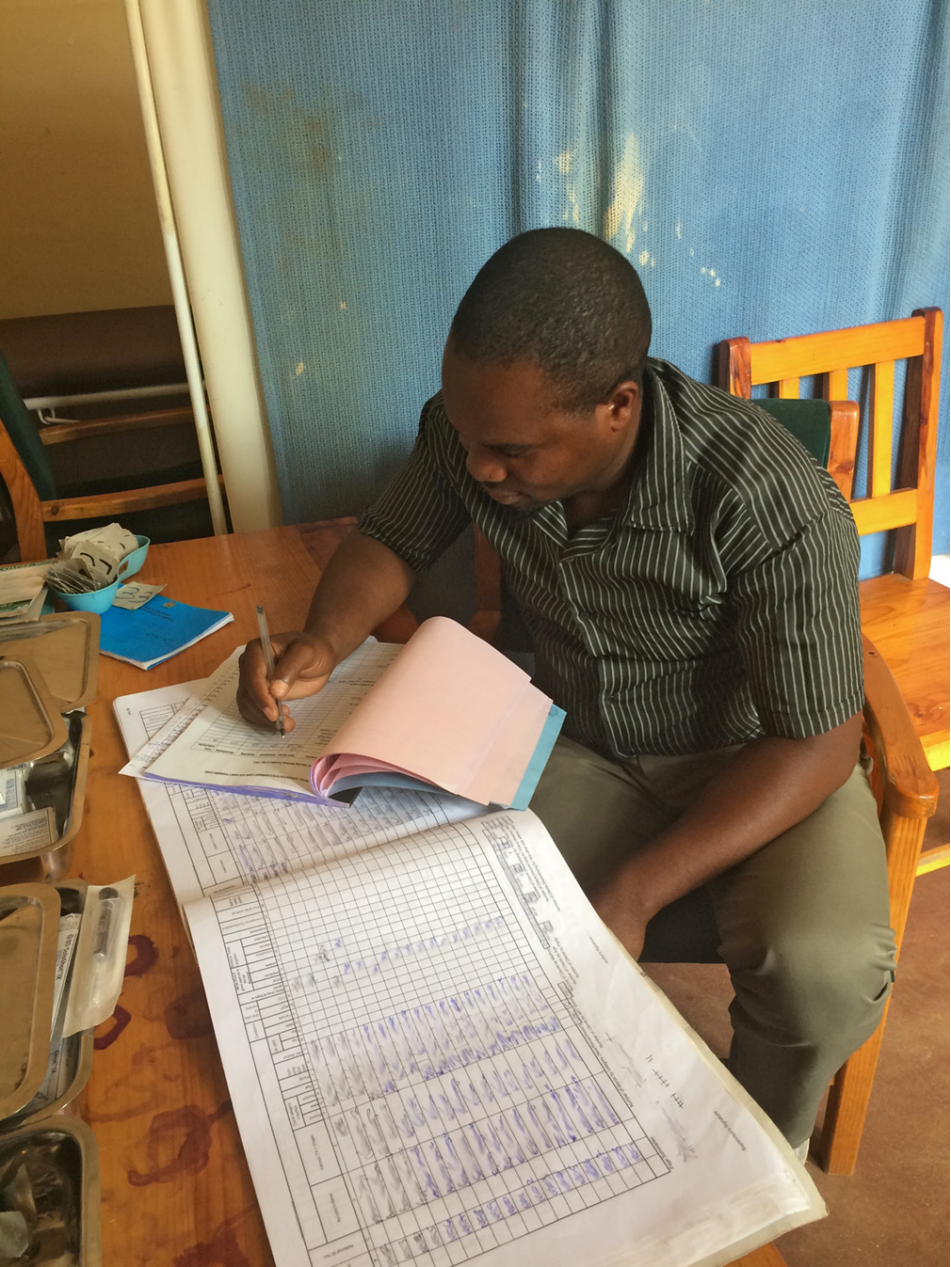
A health surveillance assistant in Malawi works to summarize his client information on community-based access to injectable for monthly reporting. ©2017 Jill M. Peterson/FHI 360

None of the people we interviewed could recall cases of infection or accidental needle sticks associated with CBA2I. Thus, it is not something for which data are regularly recorded. The officials we interviewed agreed that clinical problems should first be managed through the supervisory chain of command and then through supervisor visits as needed. This response influenced how we developed the indicator and guidance related to reportable incidents, such that health workers should be instructed on the proper clinical channel but also be given a place to record such incidents so that occurrences can be quantified.

Based on the case studies, we refined the indicators developed during the technical consultation and gained a stronger sense of what was reasonable for an M&E program to collect. We considered which indicators were already being collected, what information was available through registers or could be collected through registers, and what might be better collected through special studies or research evaluations. We also saw where programs commonly had gaps and ensured that our indicators would help programs fill them. Lastly, we considered responses from interviewees describing data they *wished* they had to better inform their management of CBA2I.

Case studies helped refine the indicators and highlighted the information that an M&E program could reasonably collect.

## DISCUSSION

The goal of our process was to develop a consensus list of CBA2I indicators and related guidance documents to strengthen CBA2I programs, resulting in increased access to and quality of family planning services. Data from the literature, global experts, and country practices provided a strong foundation for the resultant guidance and indicators. The guidance is intended for use by governments and programs or projects aiming to implement or improve CBA2I programs, and specifically, the M&E of those programs.

We recognize that 18 indicators for a subset of a family planning program might seem excessive. Within those 18 indicators, however, we have listed separately the data points needed as numerators and denominators in calculating percentages. For example, rather than listing just 1 indicator for “the proportion of CHWs certified to inject contraception,” in the full list we have included the data needed to calculate this proportion as a separate indicator (e.g., Indicator 1.1: number of CHWs trained in providing injectable contraception). In addition, many of the indicators can be used beyond CBA2I responsibilities to monitor CHW programs more generally. For example, the number of mobilization events or counseling sessions would provide general programmatic information in addition to data on CBA2I. Collectively, the 18 indicators provide a comprehensive view of a community-based family planning program with specificity for CBA2I.

For additional refinement, programs are encouraged to further disaggregate the indicators. For example, if more than 1 cadre of CHWs in a given country is providing CBA2I, the program may wish to disaggregate by cadre. In our case-study countries, CHWs were already collecting data on the number of new versus continuing users of all methods; these numbers could also be considered in the management of a CBA2I program. If programs have the resources for more in-depth measurement, they may also want to consider reasons for discontinuation, although given already heavy reporting burdens for CHWs, this topic may also be examined through a subject-specific research study.

Since our release of the guidance in March 2018, the Punjab government in Pakistan has begun providing targeted M&E using this guidance, the indicators, and the related job aids. In addition, the guidance and indicators were used in Malawi as its self-injection program was scaled up. In Malawi, we were able to see firsthand how the indicators were adapted for the local context and to the self-injection program. In some cases, the technical working group tasked with drafting M&E indicators adopted the indicators verbatim; in others, they adapted them to conform to available data sources and programmatic characteristics. By understanding the rationale behind the various indicators, program managers can get a comprehensive view of effective M&E of CBA2I and then adapt the indicators to conform to their specific contexts. The guidance, indicators, and job aids were also included in the 2018 *Community Health Worker Provision of Injectable Contraception: An Implementation Handbook*.^10^

## CONCLUSIONS

The 3-stage process we used to develop indicators and guidance for M&E of CBA2I allowed us to incorporate findings from published literature, the knowledge of experts in the field, and real-world, on-the-ground experience. By triangulating these 3 sources, we have developed a consensus list of M&E indicators as well as supporting guidance, including job aids, that will strengthen CBA2I programs and their ability to follow WHO recommendations. The guidance, an executive summary, and case study summaries can be found at https://www.k4health.org/toolkits/cba2i/step-8-document-processes-and-outcomes and at https://www.fhi360.org/resource/community-based-access-injectable-contraceptives-cba2i-select-resources.

## Notes

**Peer Reviewed**

**Funding:** The Pfizer Foundation

**Disclaimer:** This report and all recommendations and guidance contained herein are solely the product and responsibility of FHI 360.
